# Supplementary material for: Rehabilitation interventions to modify physical frailty in adults before lung transplantation: a systematic review protocol
Source: BMJ Open. 2024 Apr 3;14(4):e078561. doi: 10.1136/bmjopen-2023-078561 (PMC11146394; doi:10.1136/bmjopen-2023-078561)
Supplement: Supplementary data [file bmjopen-2023-078561supp001.pdf]

Supplementary Materials – Database search strategies

Search strategy for World Health Organisation—International clinical trials registration platform	
**Number of search**	**Search term**
1	Frail*
2	Lung transplant* AND (candidate OR wait* OR list)
3	Exercise OR prehab* OR rehab*
4	1 AND 2 AND 3

Search strategy for ClinicalTrials.gov	
**Number of search**	**Search term**
Condition or disease	Frail OR frailty OR frailness
	Lung transplant OR lung transplantation OR transplantation of lung OR transplant OR grafting procedure OR transplantation OR transplanted OR transplanting
	Lung OR pulmo*
	Rehabilitation OR rehab OR rehabilitative
Study Type	All studies
Study Results	All studies
Eligibility Criteria	Adult (18-64)<br>Sex (all)

Search strategy for CINAHL	
**Number of search**	**Search term**
1	TI lung transplant* OR AB lung transplant*
2	MH (“lung transplantation+”)
3	1 OR 2
4	TI (wait* or candidate* or pre op* or await*) OR AB (wait* or candidate* or pre op* or await*)
5	(MH “waiting lists”) OR (MH “pretransplantation period”)
6	(MH “Preoperative care+”)
7	4 OR 5 OR 6
8	TI (exercise* or rehab* or prehab*) OR AB (exercise* or rehab* or prehab*)
9	(MH “rehabilitation+”)
10	8 OR 9
11	3 AND 7 AND 10

Search strategy for Cochrane	
**Number of search**	**Search term**
1	(lung transplant*):ti,ab,kw (Word variations have been searched)
2	MeSH descriptor: [Lung Transplantation] explode all trees
3	1 OR 2
4	((wait* or candidate* or pre op* or pre-op* or await*)):ti,ab,kw (Word variations have been searched)
5	MeSH descriptor: [Waiting Lists] explode all tree
6	MeSH descriptor: [Preoperative Exercise] explode all trees
7	MeSH descriptor: [Preoperative Care] this term onl
8	4 OR 5 OR 6 OR 7
9	((exercis* or rehab* or prehab*)):ti,ab,kw (Word variations have been searched)
10	MeSH descriptor: [Rehabilitation] explode all trees
11	9 OR 10
12	3 AND 8 AND 11

Search strategy for EMBASE (OVID)	
**Number of search**	**Search term**
1	lung transplant*.ti,ab
2	exp Lung Transplantation/
3	1 or 2
4	(wait* or candidate* or pre op* or pre-op* or await*).ti,ab.
5	Waiting Lists/
6	Preoperative Exercise/ or Preoperative Care/
7	4 or 5 or 6
8	(exercis* or rehab* or prehab*).ti,ab.
9	exp Rehabilitation/
10	8 or 9
11	3 AND 7 AND 10
12	limit 11 to (yr="1980 -Current" and (adult <18 to 64 years> or aged <65+ years>))
