# Supplementary material for: Rehabilitation interventions to modify physical frailty in adults before lung transplantation: a systematic review protocol
Source: BMJ Open. 2024 Apr 3;14(4):e078561. doi: 10.1136/bmjopen-2023-078561 (PMC11146394; doi:10.1136/bmjopen-2023-078561)
Supplement: Author's manuscript [file bmjopen-2023-078561.draft_revisions.pdf]

BMJ Open is committed to open peer review. As part of this commitment we make the peer review history of every article we publish publicly available.

When an article is published we post the peer reviewers' comments and the authors' responses online. We also post the versions of the paper that were used during peer review. These are the versions that the peer review comments apply to.

The versions of the paper that follow are the versions that were submitted during the peer review process. They are not the versions of record or the final published versions. They should not be cited or distributed as the published version of this manuscript.

BMJ Open is an open access journal and the full, final, typeset and author-corrected version of record of the manuscript is available on our site with no access controls, subscription charges or pay-per-view fees (http://bmjopen.bmj.com).

If you have any questions on BMJ Open's open peer review process please email info.bmjopen@bmj.com

BMJ Open

Rehabilitation interventions to modify physical frailty in adults before lung transplantation: A systematic review protocol.

Journal:	*BMJ Open*
Manuscript ID	bmjopen-2023-078561
Article Type:	Protocol
Date Submitted by the Author:	04-Aug-2023
Complete List of Authors:	McGarrigle, Laura; Manchester University NHS Foundation Trust, Cardiothoracic Transplantation; National Institute for Health and Care Research Applied Research Collaboration Greater Manchester<br>Norman, Gill; University of Manchester, Division of Nursing, Midwifery & Social Work; National Institute for Health and Care Research Applied Research Collaboration Greater Manchester<br>Hurst, Helen; University of Salford, School of Health and Society; Northern Care Alliance NHS Foundation Trust, Renal, Salford Royal Hospital<br>Todd, Chris; University of Manchester, School of Health Sciences; National Institute for Health and Care Research Applied Research Collaboration Greater Manchester
Keywords:	Transplant medicine < INTERNAL MEDICINE, Physical Therapy Modalities, REHABILITATION MEDICINE, Cardiothoracic surgery < SURGERY, Transplant surgery < SURGERY
Note: The following files were submitted by the author for peer review, but cannot be converted to PDF. You must view these files (e.g. movies) online.	
Supplementary material [search strategies].pub	

SCHOLARONE™
Manuscripts

Rehabilitation interventions to modify physical frailty in adults before lung transplantation: A systematic review protocol.

ABSTRACT

Introduction: Lung transplantation is the gold standard treatment for end-stage lung disease for a small group of patients meeting strict acceptance criteria after optimal medical management has failed. Physical frailty is prevalent in lung transplant candidates and has been linked to worse outcomes both on the waiting list and postoperatively. Exercise has been proven to be beneficial in optimising exercise capacity and quality of life in lung transplant candidates but its impact on physical frailty is unknown. This review aims to assess the effectiveness of exercise interventions in modifying physical frailty for adults awaiting lung transplantation.

Methods and Analysis: This protocol was prospectively registered on the PROSPERO database. We will search 4 databases plus trials registries to identify primary studies including adult candidates for lung transplantation undertaking exercise interventions and assessing outcomes pertaining to physical frailty. Studies must include at least ten participants. Article screening will be performed by two researchers independently at each stage. Extraction will be performed by one reviewer and checked by a second. The risk of bias in studies will be assessed by two independent reviewers using tools appropriate for the research design of each study; where appropriate we will use Cochrane risk of bias 2 or ROBINS-I. At each stage of the review process discrepancies will be resolved through consensus or consultation with

For peer review only - http://bmjopen.bmj.com/site/about/guidelines.xhtml

For peer review only

a third reviewer. Meta-analyses of frailty outcomes, will be performed if possible and appropriate as will pre-specified subgroup and sensitivity analyses. Where we are unable to perform meta-analysis we will conduct narrative synthesis following SWiM guidance. The review will be reported using the PRISMA Checklist.

Ethics and Dissemination: No ethical issues are predicted due to the nature of this study. Dissemination will occur via conference abstracts, professional networks, peer reviewed journals and patient support groups.

Registration Details: PROSPERO registration number CRD42022363730.

Strengths and Limitations of this study

- Rigorous systematic review methods at all stages of the review combined with clinical expertise will allow us to produce a reliable first synthesis of the evidence for the effectiveness of rehabilitation in lung transplant candidates for physical frailty.
- A comprehensive search for relevant studies from multiple databases and other sources will allow us to identify relevant studies wherever published. The exclusion of non-English language studies is a limitation of this study but we will list these studies where we identify them.
- We will search for literature pertaining to "lung transplant candidates", those on the "waiting list" or people "being assessed for lung transplant". We have developed our search in consultation with an information specialist and it is likely to identify all studies where lung transplant candidates are a substantial proportion of the patient group. However it is not feasible to review every study of individuals with chronic lung

For peer review only - http://bmjopen.bmj.com/site/about/guidelines.xhtml

disease, particularly where data are unstratified by disease severity or where the authors do not identify transplant candidates. It is therefore possible that we may miss some studies with some relevant data but this is unlikely to substantively impact the review outcomes.

- Using outcomes as a key criterion for inclusion risks missing some relevant studies due to the potential for reporting bias. To mitigate this, we will attempt to contact authors of all otherwise relevant studies to establish if any further outcomes were assessed but not reported and, where possible, obtain relevant data.

For peer review only

INTRODUCTION

Lung Transplantation

Lung transplantation (LTx) involves surgically replacing diseased organs in individuals with advanced respiratory failure due to a range of lung diseases including chronic obstructive pulmonary disease (COPD), interstitial lung diseases (ILD), pulmonary arterial hypertension (PAH) and cystic fibrosis (CF). Poor lung function is associated with reduced exercise tolerance, dyspnoea and disability. The terminal stages of these conditions have a profound impact upon individual's physical function and quality of life. Lung transplantation is a well-established therapy for chronic lung disease in a very specific population who meet the

For peer review only - http://bmjopen.bmj.com/site/about/guidelines.xhtml

stringent, internationally accepted criteria(1). The strict criteria are necessary to optimise outcomes and protect from inappropriate allocation of such a scare resource. On average, there are around 350 adults on the active lung transplant waiting list in the UK. In the ten years prior to the COVID-19 pandemic, the mean number of lung transplants performed annually in the UK was 178 (±18)(2).

Frailty

Frailty is a state characterised by lack of physiological reserve and increased vulnerability to stressors and is common in chronic end-stage lung disease (3,4), particularly in those referred for lung transplants (5). There has been a significant rise in the proportion of lung transplant recipients aged over 65 years (6), and increasing age is an independent risk factor for both poor outcomes after LTx (7) and increased incidence of frailty (8).

The two main models conceptualising frailty are the phenotypic and cumulative deficit models. The phenotypic model is more commonly used in LTx and considers frailty through five characteristics; shrinking (weight loss), weakness, exhaustion, slowness and low physical activity(9). The cumulative deficit model conceptualises frailty as an accumulation of symptoms, comorbidities, diseases and health deficiencies. The greater the number of “deficits”, the higher the frailty of the individual (10). Although previously linked to decreased survival after LTx (11), the validity of the latter model in predicting other LTx outcomes is unclear (5).

Although psychosocial, nutritional and physical impairments impact post-LTx outcomes (1), the clinical operationalisation of frailty measurement in LTx relates overwhelmingly to the physical domain (5,12,13). This may be due to the feasibility of data collection, the younger age of LTx candidates or the complexity associated with cumulative deficit models (12,14).

For peer review only - http://bmjopen.bmj.com/site/about/guidelines.xhtml

The most commonly reported phenotypic frailty measures collected in clinical practice prior to LTx include the Short Physical Performance Battery (SPPB), and the Fried Frailty Phenotype (FFP) (13,15).

Research studies in LTx reflect this phenotypic approach. Although conceptualisation and investigation of biomarkers and their links to frailty (16) (including in patients with lung disease) has recently been undertaken, the field has lacked established markers or cut-offs, preventing their routine use in the evaluation of frailty for LTx (5,12,13). The recent development of a LTx specific frailty scale, the Lung Transplant Frailty Scale (LT-FS) and its validation in 342 LTx candidates may help to resolve this (13). This uses body composition (skeletal muscle mass and percentage body fat), research-grade serum biomarkers and clinical laboratory blood markers in addition to measurement of physical function (SPPB and FFP). All models of the scale displayed superior predictive validity for short term mortality and removal from the LTx waiting list than the FFP or SPPB alone, but long term outcomes have not yet been evaluated.

Previous reviews have identified physical frailty as being detrimental to morbidity and mortality both before and after LTx (17). Physical frailty is also associated with an increased risk of readmission after LTx (18). Transplant teams experience the challenges of identifying patients with the physical and psychological reserve necessary to survive such a demanding peri and postoperative period and to thrive long-term (1,12). This challenge has been exacerbated by the lack of consensus on the optimal assessment tools for frailty in this field (1), which the LT-FS may help to resolve (13). Despite the current lack of consensus on tool utility, it is widely agreed that further work is required to establish the effectiveness of

For peer review only - http://bmjopen.bmj.com/site/about/guidelines.xhtml

interventions to modify physical frailty, improve candidate selection and LTx outcomes (1,13,19).

Exercise and Rehabilitation Prior to Lung Transplantation

Rehabilitation for individuals with lung conditions usually takes the form of pulmonary rehabilitation; an evidence-based programme of exercise interventions and education. It aims to reduce dyspnoea, optimise functional capacity, increase participation, and reduce healthcare costs through exacerbations and hospital admissions (20). Rehabilitation whilst on the waiting list (sometimes known as prehabilitation) is recommended for all LTx candidates(1). Rehabilitation after LTx is also a vital component of the recovery programme after surgery and ICU/hospital stay (21). The course of the SARS COVID-19 pandemic has seen many pulmonary rehabilitation schemes develop virtual or telephone offers alongside a face-to-face programme. Technological advances have also led to the development of digital alternatives (22).

Potential Impact of the Intervention

Advancing lung disease impacts on exercise capacity and accelerates muscle atrophy. Rehabilitation prior to LTx has been shown to improve exercise capacity and quality of life with some evidence of increases in muscle strength (21). Exercise has the potential to contribute to an increase in muscle strength, balance and physical activity which in turn impacts on physical frailty (23). Pulmonary rehabilitation has been shown to reverse frailty in the short term in individuals with COPD when assessed using the FFP (24).

Why it is Important to do this Review

For peer review only - http://bmjopen.bmj.com/site/about/guidelines.xhtml

Professionals in the transplant community are increasingly measuring frailty in attempts to evaluate surgical suitability and weigh the risks and potential benefits of LTx for older individuals. There is emerging evidence linking frailty to reduced quality of life and mortality on the lung transplant waiting list and postoperative adverse outcomes including increased length of hospital stay, readmission, disability and worse health related quality of life (5). Frailty is therefore a significant concern for transplant programmes.

The aim of LTx is to improve survival, function and quality of life of the recipient, whilst considering the ethical elements of who will benefit from such a limited pool of donor organs (12). Understanding and targeting reversible components of frailty has the potential to improve physical condition ahead of major surgery, reduce waiting list mortality and potentially impact post-operative outcomes and maximise benefit from LTx (4,12,25). Identifying the best strategies to improve frailty prior to transplantation has been highlighted as an area that requires investigation (4,5,13).

Previous systematic reviews have documented the beneficial effect of rehabilitation on exercise capacity and quality of life prior to a lung transplant (21,26). However, there are no prior reviews specifically addressing interventions in modifying physical frailty in this population despite the significance of the problem and link to poor outcomes (17).

Aims and Objectives

This systematic review aims to evaluate the impact of exercise interventions in modifying physical frailty for adults awaiting lung transplantation. We also aim to identify any harms that occur as a result of an exercise intervention.

For peer review only - http://bmjopen.bmj.com/site/about/guidelines.xhtml
For peer review only

METHODS AND ANALYSIS

Study Design

This study will be a systematic review with methodology following guidance from the Cochrane Handbook of Systematic Reviews (27). It will be reported in accordance with the Preferred Reporting Items for Systematic Reviews and Meta-Analyses checklist (PRISMA) (28). Any important amendments will be documented on Prospero.

Inclusion and Exclusion Criteria

Types of Studies:

Preliminary searches indicated a very few randomised controlled trials (RCT) and non-randomised controlled studies, which we initially planned to include. We therefore widened the inclusion criteria to original primary research studies with any design, including those without controls, with more than 10 participants; this amendment was registered on Prospero. We therefore anticipate including randomised and non-randomised trials, cohort studies and case series. Cross-sectional studies may be eligible if they assess frailty following an intervention. We will not include evidence syntheses, commentaries, case studies, and non-systematic narrative reviews.

Types of Participants:

We will include studies of adults over the age of 18, on a waiting list (candidates) for a single or double lung transplant. Individuals with any underlying lung disease who have met the criteria to be accepted onto the waiting list at a lung transplant centre, where frailty has been assessed will be included. There are well established and accepted internationally agreed criteria for lung transplantation (1). We will include studies involving candidates for primary

For peer review only - http://bmjopen.bmj.com/site/about/guidelines.xhtml

LTx or subsequent repeat LTx procedures. LTx performed by any surgical incision site will be included. Studies of candidates listed for a multi-organ transplant in the same surgical procedure (including but not limited to, concurrent heart and lung or lung and liver) will be excluded unless data is provided for lung recipients only or multi-organ candidates comprise under 25% of study We will exclude studies of people completing interventions whilst receiving extra corporeal membrane oxygenation (ECMO) as a bridge to transplant; they are restricted to interventions within the intensive care unit which is not the focus of this review.

Types of Intervention

We will include any formal physical exercise or physical activity delivered prescribed under professional guidance which includes formal evaluation of outcomes. The intervention may be supervised or unsupervised, face to face or virtual and performed in any setting (community or hospital). There is no minimum length or intensity of intervention. The intervention may be single (one form of exercise) or multimodal (for example a combination of strength and endurance training). The intervention must contain an exercise or physical activity component. Physical activity is defined as any bodily movement produced by skeletal muscles that results in energy expenditure. Exercise is defined as a subset of physical activity that is planned, structured and repetitive and has an objective of the improvement or maintenance of physical fitness (29).

Types of Comparators

We will accept any comparator or no comparator. Where studies include comparison groups we anticipate identifying comparisons of rehabilitation with one or more of the following: no

For peer review only - http://bmjopen.bmj.com/site/about/guidelines.xhtml

intervention, “usual care”, advice only, or an alternative intervention which may or may not meet our intervention criteria. The specific rehabilitation intervention should be the only systematic difference between the groups.

Outcomes:

We will consider the following physical frailty measures as primary outcomes: measures of Phenotypic frailty (FFP) or cumulative deficit frailty models (e.g. Clinical Frailty Scale, Electronic Frailty Index,).

We will also include surrogate markers of frailty e.g. SPPB, sarcopenia via hand grip dynamometry, quadriceps force, CT scan (muscle cross sectional area), muscle strength testing of upper of lower limb: manual or non-manual (e.g. 1 rep max), sit to stand testing and objective assessments of balance.

Studies must include at least one direct or indirect measure of frailty in order to be eligible for inclusion. Where no relevant outcome is reported we will attempt to contact authors of all otherwise relevant studies to establish if any further outcomes were assessed but not reported and, where possible, obtain relevant data. We will distinguish between studies excluded after confirmation that no relevant outcomes were assessed and those where this confirmation was not possible.

Where studies report a relevant primary outcome we will consider the following as secondary outcomes: Mortality (on waiting list or post-operatively), hospital/ICU length of stay, health-related quality of life measures. Any adverse events reported during interventions will be recorded.

For peer review only - http://bmjopen.bmj.com/site/about/guidelines.xhtml

Search Strategies

Literature search strategies have been developed using medical subject headings (MeSH) related to rehabilitation, exercise and lung transplant candidates with support from an experienced medical librarian. Trials will be identified from searches of the following databases: MEDLINE (Ovid) 1980 to date, EMBASE (Ovid) 1980 to date, CINHAL Plus (EBSCO) 1980 to date, Cochrane Central Register of Controlled Trials (CENTRAL), the Cochrane Library, trials registries (ClinicalTrials.gov and the WHO trials portal). All databases will be searched from 1980 to the present. The success of lung transplantation was established only after the discovery and introduction of the immunosuppressive agent cyclosporine which became accepted practice in the early 1980s (30). A draft search strategy for MEDLINE is given in Table 1 with this strategy being adopted and modified for other databases (see supplementary material).

We will also search the references of all identified included studies and of identified systematic or scoping reviews.

Table 1. Ovid MEDLINE(R) Search strategy

**Number of search**	**Search term**
1	lung transplant*.ti,ab.
2	exp Lung Transplantation/
3	1 OR 2
4	(wait* or candidate* or pre op* or pre-op* or await*).ti,ab.
5	Waiting Lists/
6	Preoperative Exercise/ or Preoperative Care/
7	4 or 5 or 6
8	(exercis* or rehab* or prehab*).ti,ab.

For peer review only

For peer review only - http://bmjopen.bmj.com/site/about/guidelines.xhtml

9	exp Rehabilitation/
10	8 OR 9
11	3 AND 7 AND 10
12	limit 11 to (yr="1980 -Current" and "all adult (19 plus years)")

Selecting Studies

Following deduplication in Endnote, title and abstract and full text screening will be performed by two independent reviewers (LM and GN) against the pre-defined inclusion criteria using Rayyan software (31). Any discrepancies between reviewers will be resolved by consensus or consultation with a third reviewer (HH or CT). Any non-English language studies will be retained and listed for reference but not included in the synthesis process. This will enhance transparency, mitigate language publication bias and maps the extent of missing evidence in our results.

Data Extraction and Management

Two authors will pilot and agree a data extraction form. Data extraction will be completed by one author (LM) and checked by a second (GN). Any discrepancy will be reconciled by consensus or a third author (HH or CT). The data extraction form will be based on the information collated in Table 2.

**Table 2: Data extraction plan.**	
	**Data to be extracted**
**Study Design**	Design (e.g. RCT)<br>Date of study period<br>Country and setting e.g. single site or multi-centre study

For peer review only - http://bmjopen.bmj.com/site/about/guidelines.xhtml

	Duration of follow-up<br>Sample size calculation<br>Ethical approval and (methods of) informed consent
**Participants**	Inclusion/Exclusion Criteria<br>Characteristics for each group including:<br>Number of participants<br>Age (Mean (s.d.))<br>Ratio of male:female<br>Disease/diagnosis<br>Co-morbidities<br>Primary transplant (or repeat transplant after graft failure); number/percentage per group.<br>Characteristics that stratify health opportunities and outcomes (PROGRESS-plus) (32)
**Interventions**	Based on TIDieR: Template for Intervention Description and Replication Checklist (33).<br>Type of exercise component/other intervention in each group<br>Frequency, intensity and duration of intervention (including how this was prescribed)<br>Location of intervention delivery (e.g. Home, hospital, community, gym)<br>Who delivered the intervention<br>Mode of intervention delivery (e.g. Face to face, telephone, video call)<br>Tailoring / modification<br>Adherence measured?
**Outcomes and measures of effect**	Definition for each outcome alongside minimal clinically important difference (MCID) where available, scale limits and direction of benefit.<br>Time points measured.<br>Validation of tool<br>Outcomes: For each group and each outcome at each time point we will extract the number of participants with data in each group and the following data:

For peer review only - http://bmjopen.bmj.com/site/about/guidelines.xhtml

	Continuous outcomes: mean with s.d. for each group. Medians with IQR will be extracted where reported. Mean differences or standardised mean differences with 95% CI will be extracted where these are the only reported data. P values will be extracted in the absence of other outcome data as will descriptive reporting of results.<br>Dichotomous outcomes: number in each group with event. For adverse events types of adverse events will also be reported. Relative risks or odds ratios with 95% CI will be extracted where these are the only reported data.<br>Time to event data: hazard ratios or data to calculate these.
**Other**	Other relevant details of study design or intervention.<br>Funding Source<br>Conflicts of interest<br>Recruitment failure<br>Patient and public involvement or engagement

Where outcomes use continuous scales of measurement (e.g. composite frailty scores, muscle cross-sectional area) we will express the results as the mean difference or, where scales or units are unclear or composites, standardised mean difference with 95% confidence intervals (CI). We will express dichotomous outcomes as risk ratios (RR) with 95% CI. Survival outcomes will be reported as hazard ratios with 95% CI. If outcomes are reported at multiple time points we will extract all the data, but will analyse primary data at the end of the intervention and at the latest time point reported.

Missing Data:

Any missing data will be requested from the original author by email and included in the review. If we are unable to gather the missing data, where possible, we will calculate or impute these (e.g. where measures of variance are not available). Where outcome data

For peer review only

For peer review only - http://bmjopen.bmj.com/site/about/guidelines.xhtml

remain missing and cannot be imputed we will use assumptions in analysing dichotomous data; that is we will assume that missing participants did not have the outcome assessed but we will explore the impact of this assumption with a sensitivity analysis. For continuous outcomes we will perform a completed case analysis and will not attempt to impute results for missing participants.

Risk of Bias Assessment

RCTs and quasi-RCTs will be assessed using the Cochrane Risk of Bias tool (RoB2) with an extension for cluster or crossover RCTs as required (27). ROBINS-I (34) or another appropriate tool will be used for non-randomised controlled studies. Other study designs (e.g. cohort and cross-sectional studies and case-series) will be assessed using an appropriate tool. Risk of bias assessments will be done by two reviewers independently and any discrepancies reconciled by a 3rd reviewer.

Data analysis - Strategy for Data Synthesis

Clinical and methodological heterogeneity will be considered initially. If the studies identified vary widely in terms of population, study design, intervention and outcomes a meta-analysis may be inappropriate and therefore a narrative synthesis of findings will be produced and reported following the Synthesis without Meta-analysis (SWiM) guidelines for narrative evidence synthesis (35). If sufficient studies are identified with low heterogeneity, statistical heterogeneity will be considered to determine if a random-effects meta-analysis can be performed.

Assessment of heterogeneity

For peer review only - http://bmjopen.bmj.com/site/about/guidelines.xhtml

We will initially assess statistical heterogeneity by visual inspection of the forest plot followed by a quantification using the I^2 statistic. (75% - 100% considerable heterogeneity; 50-90% considered to represent substantial heterogeneity; 30-60% moderate and 0-40% considered unimportant heterogeneity) (27)

Subgroup analysis

Participant heterogeneity may be investigated using subgroup analysis by disease type (COPD, CF, IPF for example). Subgroup analysis by age may be considered (18-69, and ≥70 years) as lung transplant recipients over the age of 70 years have a decreased longer term survival (36). Intervention heterogeneity may be investigated by subgroup analysis including the following potential groups: virtual and in person rehabilitation, exercise intensity, exercise type (e.g. aerobic versus resistance) or the degree of supervision (e.g. fully supervised versus non supervised). We will exercise caution in implementing these prespecified analyses and are mindful of the risks of performing multiple such analyses.

Sensitivity analysis

If appropriate we will use sensitivity analyses to explore the impact of any imputation of or assumptions about missing data. If we have performed a meta-analysis with very few studies we will conduct a fixed effects analysis to assess the impact of the random effects model in this context (37).

Summary of Findings tables.

We will present a summary of the main findings of the review in a table format including key information regarding the magnitude of effects for each outcome alongside a rating of the certainty of evidence. We will use the Grading of Recommendations Assessment,

For peer review only - http://bmjopen.bmj.com/site/about/guidelines.xhtml

Development and Evaluation working group methodology considerations (risk of bias, consistency of effect, imprecision, indirectness and publication bias) to assess the quality of the body of evidence for each prespecified outcome (38,39). We will justify all decisions to down- or up-grade the certainty of the evidence. If appropriate we will present the evidence in summary of findings tables generated using GRADEpro software (40).

Patient and Public Involvement

Local lung transplant support group attendees have been involved in some preliminary patient and public engagement work prior to the data collection for this study. Further PPI work will be completed to contribute to the production of a plain language summary and insight into dissemination strategies.

ETHICS AND DISSEMINATION

This systematic review has been registered prospectively (registration number CRD42022363730). Its findings will be submitted to international transplantation conferences and peer reviewed journals for dissemination of the results and conclusions. Results, and their implications will be shared at local and national meetings of LTx clinicians. We will also share findings with people living with chronic lung disease on the transplant waiting list and their carers via local patient support groups.

REFERENCE LIST

For peer review only

For peer review only - http://bmjopen.bmj.com/site/about/guidelines.xhtml

1. Leard LE, Holm AM, Valapour M, et al. Consensus document for the selection of lung transplant candidates: An update from the International Society for Heart and Lung Transplantation. J Heart Lung Transplant. 2021;40(11):1349-79.
2. NHSBT Annual report of cardiothoracic organ transplantation [website] London (UK) 2020 [Updated 2020, cited March 2023]. Available from https://nhsbtdbe.blob.core.windows.net/umbraco-assets-corp/19874/nhsbt-annual-report-on-cardiothoracic-organ-transplantation-201920.pdf
3. Venado A, McCulloch C, Greenland JR, et al. Frailty trajectories in adult lung transplantation: A cohort study. The Journal of Heart and Lung Transplantation. 2019;38(7):699-707.
4. Kobashigawa J, Dadhania D, Bhorade S, et al. Report from the American Society of Transplantation on frailty in solid organ transplantation. American Journal of Transplantation. 2019;19(4):984-94.
5. Varughese R, Rozenberg D, Singer LG. An update on frailty in lung transplantation. Current Opinion in Organ Transplantation. 2020;25(3):274-9.
6. Valapour M, Lehr CJ, Skeans MA, et al. OPTN/SRTR 2018 Annual Data Report: Lung. American Journal of Transplantation. 2020;20(s1):427-508.
7. Courtwright A, Cantu E. Lung transplantation in elderly patients. J Thorac Dis. 2017;9(9):3346-51
8. Clegg A, Young J, Iliffe S, et al. Frailty in elderly people. Lancet. 2013;381(9868):752-62.
9. Fried LP, Tangen CM, Walston J, et al. Frailty in older adults: evidence for a phenotype. J Gerontol A Biol Sci Med Sci. 2001;56(3):M146-56.
10. Rockwood K, Mitnitski A. Frailty in Relation to the Accumulation of Deficits. The Journals of Gerontology: Series A. 2007;62(7):722-7.
11. Wilson ME, Vakil AP, Kandel P, et al. Pretransplant frailty is associated with decreased survival after lung transplantation. J Heart Lung Transplant. 2016;35(2):173-8.

For peer review only - http://bmjopen.bmj.com/site/about/guidelines.xhtml

12. Agarwal A, Neujahr DC. Frailty in lung transplantation: Candidate assessment and optimization. Transplantation. 2021;105(10):2201-12.

13. Singer JP, Christie JD, Diamond JM, et al. Development of the Lung Transplant Frailty Scale (LT-FS). J Heart Lung Transplant. 2023;42(7):892-904.

14. Spiers GF, Kunonga TP, Hall A, et al. Measuring frailty in younger populations: a rapid review of evidence. BMJ Open. 2021;11(3):e047051.

15. Snyder LD, Singer LG. Beyond the eyeball test: Measures of frailty in lung transplantation. The Journal of Heart and Lung Transplantation. 2019;38(7):708-9.

16. Hakeem FF, Maharani A, Todd C, et al. Development, validation and performance of laboratory frailty indices: A scoping review. Archives of Gerontology and Geriatrics. 2023;111:104995.

17. Montgomery E, Macdonald PS, Newton PJ, et al. Frailty in lung transplantation: a systematic review. Expert Review of Respiratory Medicine. 2020;14(2):219-27.

18. Courtwright AM, Zaleski D, Gardo L, et al. Causes, Preventability, and Cost of Unplanned Rehospitalizations Within 30 Days of Discharge After Lung Transplantation. Transplantation. 2018;102(5):838-44

19. Wilson ME, Vakil AP, Kandel P, et al. Pretransplant frailty is associated with decreased survival after lung transplantation. J Heart Lung Transplant. 2016;35(2):173-8.

20. Holland AE, Cox NS, Houchen-Wolloff L, et al. Defining Modern Pulmonary Rehabilitation. An Official American Thoracic Society Workshop Report. Ann Am Thorac Soc. 2021;18(5):e12-e29.

21. Hume E, Ward L, Wilkinson M, et al. Exercise training for lung transplant candidates and recipients: A systematic review. European Respiratory Review. 2020;29(158):1-19.

22. Singer JP, Soong A, Bruun A, et al. A mobile health technology enabled home-based intervention to treat frailty in adult lung transplant candidates: A pilot study. Clin Transplant. 2018;32(6):e13274.

For peer review only - http://bmjopen.bmj.com/site/about/guidelines.xhtml

23. Angulo J, El Assar M, Álvarez-Bustos A, et al. Physical activity and exercise: Strategies to manage frailty. Redox Biol. 2020;35:101513.

24. Maddocks M, Kon SSC, Canavan JL, et al. Physical frailty and pulmonary rehabilitation in COPD: a prospective cohort study. Thorax. 2016;71(11):988-95.

25. Rozenberg D, Orsso CE, Chohan K, et al. Clinical outcomes associated with computed tomography-based body composition measures in lung transplantation: a systematic review. Transpl Int. 2020;33(12):1610-25.

26. Hoffman M, Chaves G, Ribeiro-Samora GA, et al. Effects of pulmonary rehabilitation in lung transplant candidates: a systematic review. BMJ open. 2017 Feb 1;7(2):e013445.

27. Higgins JPT, Thomas J, Chandler J, et al. (editors)[website]. Cochrane Handbook for Systematic Reviews of Interventions version 6.3 (updated February 2022). Cochrane, 2022. Available from www.training.cochrane.org/handbook

28. Page MJ, McKenzie JE, Bossuyt PM, et al. The PRISMA 2020 statement: an updated guideline for reporting systematic reviews. BMJ. 2021;372:n71.

29. Casperson CJ, Powell KE, Christenson GM. Physical activity, exercise and physical fitness: Definitions and distinctions for health-related research. Public Health Reports. 1985;100(2):126-31.

30. Venuta F, Van Raemdonck D. History of lung transplantation. J Thorac Dis. 2017;9(12):5458-71.

31. Ouzzani M, Hammady H, Fedorowicz Z, et al. Rayyan—a web and mobile app for systematic reviews. Systematic Reviews. 2016;5(1):210.

32. O'Neill J, Tabish H, Welch V, et al. Applying an equity lens to interventions: using PROGRESS ensures consideration of socially stratifying factors to illuminate inequities in health. Journal of Clinical Epidemiology. 2014;67(1):56-64.

For peer review only - http://bmjopen.bmj.com/site/about/guidelines.xhtml

33. Hoffmann TC, Glasziou PP, Boutron I, et al. Better reporting of interventions: template for intervention description and replication (TIDieR) checklist and guide. BMJ : British Medical Journal. 2014;348:g1687.

34. Sterne JAC, Hernán MA, Reeves BC, et al. ROBINS-I: a tool for assessing risk of bias in non-randomised studies of interventions. BMJ. 2016;355:i4919.

35. Campbell M, McKenzie JE, Sowden A, et al. Synthesis without meta-analysis (SWiM) in systematic reviews: reporting guideline. BMJ. 2020;368:l6890.

36. Hayanga AJ, Aboagye JK, Hayanga HE, et al. Contemporary analysis of early outcomes after lung transplantation in the elderly using a national registry. J Heart Lung Transplant. 2015;34(2):182-8.

37. Bender R, Friede T, Koch A, et al. Methods for evidence synthesis in the case of very few studies. Research Synthesis Methods. 2018;9(3):382-92.

38. Guyatt G, Oxman AD, Akl EA, et al. GRADE guidelines: 1. Introduction—GRADE evidence profiles and summary of findings tables. Journal of Clinical Epidemiology. 2011;64(4):383-94.

39. Schünemann H, Brożek J, Guyatt G, Oxman A. GRADE handbook for grading quality of evidence and strength of recommendations. guidelinedevelopment.org/handbook2013

40. GRADEpro GDT: GRADEpro Guideline Development Tool [Software]. McMaster University and Evidence Prime, 2022. Available from gradepro.org

For peer review only - http://bmjopen.bmj.com/site/about/guidelines.xhtml

ACKNOWLEDGEMENTS AND STATEMENTS OF FINANCIAL SUPPORT

LM holds a part-time predoctoral fellowship funded by the National Institute for Health and Care Research Applied Research Collaboration Greater Manchester (NIHR ARC-GM)(Grant award number NIHR200174). CT is a CI and partially funded and GN is wholly funded by NIHR ARC-GM. The views expressed in this publication are those of the authors and not necessarily those of the National Institute for Health and Care Research or the Department of Health and Social Care or its partner organisations.

Contributions: LM had the idea for the review, wrote the first draft of the protocol and designed the search strategy; GN, HH and CT advised on the protocol, edited and commented substantively on the protocol; all authors approved the manuscript prior to submission.

Competing Interests Declaration: The authors have no competing interests to declare.

Acknowledgements: The authors thank Bethan Morgan (librarian at Manchester University NHS Foundation Trust) for her support with electronic search strategies for this review.

For peer review only

For peer review only

For peer review only - http://bmjopen.bmj.com/site/about/guidelines.xhtml

For peer review only

For peer review only - http://bmjopen.bmj.com/site/about/guidelines.xhtml

PRISMA-P (Preferred Reporting Items for Systematic review and Meta-Analysis Protocols) 2015 checklist: recommended items to address in a systematic review protocol*

Section and topic	Item No	Checklist item	Information reported?	Page number
**ADMINISTRATIVE INFORMATION**				
Title:		Rehabilitation interventions to modify physical frailty in adults before lung transplantation: A systematic review protocol		
Identification	1a	Identify the report as a protocol of a systematic review	Yes	1
Update	1b	If the protocol is for an update of a previous systematic review, identify as such	n/a	n/a
Registration	2	If registered, provide the name of the registry (such as PROSPERO) and registration number	Yes	2
Authors:				
Contact	3a	Provide name, institutional affiliation, e-mail address of all protocol authors; provide physical mailing address of corresponding author	Yes	Submitted to journal
Contributions	3b	Describe contributions of protocol authors and identify the guarantor of the review	Yes	22
Amendments	4	If the protocol represents an amendment of a previously completed or published protocol, identify as such and list changes; otherwise, state plan for documenting important protocol amendments	Yes (plan for reporting amendments)	8
Support:				
Sources	5a	Indicate sources of financial or other support for the review	Yes	22
Sponsor	5b	Provide name for the review funder and/or sponsor	Yes	22
Role of sponsor or funder	5c	Describe roles of funder(s), sponsor(s), and/or institution(s), if any, in developing the protocol	Yes	22
**INTRODUCTION**				
Rationale	6	Describe the rationale for the review in the context of what is already known	Yes	6-7
Objectives	7	Provide an explicit statement of the question(s) the review will address with reference to participants, interventions, comparators, and outcomes (PICO)	Yes	7
**METHODS**				
Eligibility criteria	8	Specify the study characteristics (such as PICO, study design, setting, time frame) and report characteristics (such as years considered, language, publication status) to be used as criteria for eligibility for the review	Yes	8-11
Information sources	9	Describe all intended information sources (such as electronic databases, contact with study authors, trial registers or other grey literature sources) with planned dates of coverage	Yes	11,14.

For peer review only

Search strategy	10	Present draft of search strategy to be used for at least one electronic database, including planned limits, such that it could be repeated	Yes	11 (and supplementary info)
Study records:				
Data management	11a	Describe the mechanism(s) that will be used to manage records and data throughout the review	Yes	12
Selection process	11b	State the process that will be used for selecting studies (such as two independent reviewers) through each phase of the review (that is, screening, eligibility and inclusion in meta-analysis)	Yes	12
Data collection process	11c	Describe planned method of extracting data from reports (such as piloting forms, done independently, in duplicate), any processes for obtaining and confirming data from investigators	Yes	12,14
Data items	12	List and define all variables for which data will be sought (such as PICO items, funding sources), any pre-planned data assumptions and simplifications	Yes	12-16 Table 2
Outcomes and prioritization	13	List and define all outcomes for which data will be sought, including prioritization of main and additional outcomes, with rationale	Yes	10 Table 2 – page 13
Risk of bias in individual studies	14	Describe anticipated methods for assessing risk of bias of individual studies, including whether this will be done at the outcome or study level, or both; state how this information will be used in data synthesis	Yes	15-17
Data synthesis	15a	Describe criteria under which study data will be quantitatively synthesised	Yes	15-16
	15b	If data are appropriate for quantitative synthesis, describe planned summary measures, methods of handling data and methods of combining data from studies, including any planned exploration of consistency (such as I^2, Kendall's τ)	Yes	15-16
	15c	Describe any proposed additional analyses (such as sensitivity or subgroup analyses, meta-regression)	Yes	16
	15d	If quantitative synthesis is not appropriate, describe the type of summary planned	Yes	
Meta-bias(es)	16	Specify any planned assessment of meta-bias(es) (such as publication bias across studies, selective reporting within studies)	n/a	Selective reporting: page 10
Confidence in cumulative evidence	17	Describe how the strength of the body of evidence will be assessed (such as GRADE)	Yes	16-17

*** It is strongly recommended that this checklist be read in conjunction with the PRISMA-P Explanation and Elaboration (cite when available) for important clarification on the items. Amendments to a review protocol should be tracked and dated. The copyright for PRISMA-P (including checklist) is held by the PRISMA-P Group and is distributed under a Creative Commons Attribution Licence 4.0.**

For peer review only

From: Shamseer L, Moher D, Clarke M, Ghersi D, Liberati A, Petticrew M, Shekelle P, Stewart L, PRISMA-P Group. Preferred reporting items for systematic review and meta-analysis protocols (PRISMA-P) 2015: elaboration and explanation. BMJ. 2015 Jan 2;349(jan02 1):g7647.

For peer review only

BMJ Open

Rehabilitation interventions to modify physical frailty in adults before lung transplantation: A systematic review protocol.

Journal:	*BMJ Open*
Manuscript ID	bmjopen-2023-078561.R1
Article Type:	Protocol
Date Submitted by the Author:	11-Dec-2023
Complete List of Authors:	McGarrigle, Laura; Manchester University NHS Foundation Trust, Cardiothoracic Transplantation; National Institute for Health and Care Research Applied Research Collaboration Greater Manchester<br>Norman, Gill; University of Manchester, Division of Nursing, Midwifery & Social Work; National Institute for Health and Care Research Applied Research Collaboration Greater Manchester<br>Hurst, Helen; University of Salford, School of Health and Society; Northern Care Alliance NHS Foundation Trust, Renal, Salford Royal Hospital<br>Todd, Chris; University of Manchester, School of Health Sciences; National Institute for Health and Care Research Applied Research Collaboration Greater Manchester
**Primary Subject Heading**:	Rehabilitation medicine
Secondary Subject Heading:	Respiratory medicine
Keywords:	Transplant medicine < INTERNAL MEDICINE, Physical Therapy Modalities, REHABILITATION MEDICINE, Cardiothoracic surgery < SURGERY, Transplant surgery < SURGERY

SCHOLARONE™
Manuscripts

Rehabilitation interventions to modify physical frailty in adults before lung transplantation: A systematic review protocol.

Laura Mcgarrigle[1], Gill Norman[2], Helen Hurst[3], Chris Todd[2].

[1] Department of Cardiothoracic Transplantation, Manchester University NHS Foundation Trust, Manchester, United Kingdom.

[2] School of Health Sciences, Faculty of Biology, Medicine and Health, University of Manchester, Manchester, United Kingdom.

[3] School of Health and Society, University of Salford and Northern Care Alliance NHS Foundation Trust, Salford, United Kingdom.

Corresponding author:

Laura McGarrigle, Department of Cardiothoracic Transplantation, Wythenshawe Hospital, Southmoor Road, Manchester. M239LT. United Kingdom.

Laura.mcgarrigle@mft.nhs.uk

Tel: +44 161 2915703 or 07865065412 Fax: +44 161 291 2205

Keywords: Lung Transplantation, rehabilitation, frailty, exercise, waiting list.

Word Count: 4195

For peer review only - http://bmjopen.bmj.com/site/about/guidelines.xhtml

ABSTRACT

Introduction: Lung transplantation is the gold standard treatment for end-stage lung disease for a small group of patients meeting strict acceptance criteria after optimal medical management has failed. Physical frailty is prevalent in lung transplant candidates and has been linked to worse outcomes both on the waiting list and postoperatively. Exercise has been proven to be beneficial in optimising exercise capacity and quality of life in lung transplant candidates but its impact on physical frailty is unknown. This review aims to assess the effectiveness of exercise interventions in modifying physical frailty for adults awaiting lung transplantation.

Methods and Analysis: This protocol was prospectively registered on the PROSPERO database. We will search four databases plus trials registries to identify primary studies of adult candidates for lung transplantation undertaking exercise interventions and assessing outcomes pertaining to physical frailty. Studies must include at least ten participants. Article screening will be performed by two researchers independently at each stage. Extraction will be performed by one reviewer and checked by a second. The risk of bias in studies will be assessed by two independent reviewers using tools appropriate for the research design of each study; where appropriate we will use Cochrane risk of bias 2 or ROBINS-I. At each stage of the review process discrepancies will be resolved through consensus or consultation with a third reviewer. Meta-analyses of frailty outcomes, will be performed if possible and appropriate as will pre-specified subgroup and sensitivity analyses. Where we are unable to perform meta-analysis we will conduct narrative synthesis following SWiM guidance. The review will be reported using the PRISMA Checklist.

For peer review only - http://bmjopen.bmj.com/site/about/guidelines.xhtml

Ethics and Dissemination: No ethical issues are predicted due to the nature of this study. Dissemination will occur via conference abstracts, professional networks, peer reviewed journals and patient support groups.

Registration Details: PROSPERO registration number CRD42022363730.

Strengths and Limitations of this study

- Rigorous systematic review methods at all stages of the review combined with clinical expertise will allow us to produce a reliable first synthesis of the evidence for the effectiveness of rehabilitation in lung transplant candidates for physical frailty.
- A comprehensive search for relevant studies, with input from an information specialist, from multiple databases and other sources will allow us to identify relevant studies wherever published.
- The exclusion of non-English language studies is a limitation of this study but we will list these studies where we identify them.
- It is possible that we may miss some studies of individuals with chronic lung disease with relevant data, where the inclusion of lung transplant candidates is not specified by the authors, but this is unlikely to substantively impact the review outcomes.
- Using outcomes as a key criterion for inclusion risks missing some relevant studies due to the potential for reporting bias; to mitigate this, we will attempt to contact authors of all otherwise relevant studies to establish if any further outcomes were assessed but not reported and, where possible, obtain relevant data.

For peer review only - http://bmjopen.bmj.com/site/about/guidelines.xhtml

INTRODUCTION

Lung Transplantation

Lung transplantation (LTx) involves surgically replacing diseased organs in individuals with advanced respiratory failure due to a range of lung diseases including chronic obstructive pulmonary disease (COPD), interstitial lung diseases (ILD), pulmonary arterial hypertension (PAH) and cystic fibrosis (CF). Poor lung function is associated with reduced exercise tolerance, dyspnoea and disability. The terminal stages of these conditions have a profound impact upon individual's physical function and quality of life. LTx is a well-established therapy for chronic lung disease in a very specific population who meet the stringent, internationally accepted criteria[1]. The strict criteria are necessary to optimise outcomes and protect from inappropriate allocation of such a scare resource. On average, there are around 350 adults on the active LTx waiting list in the UK. In the ten years prior to the COVID-19 pandemic, the mean number of lung transplants performed annually in the UK was 178 (±18)[2].

Frailty

Frailty is a state characterised by lack of physiological reserve and increased vulnerability to stressors and is common in chronic end-stage lung disease [3,4], particularly in those referred for LTx [5]. There has been a significant rise in the proportion of LTx recipients aged over 65 years [6], and increasing age is an independent risk factor for both poor outcomes after LTx [7] and increased incidence of frailty [8].

The two main models conceptualising frailty are the phenotypic and cumulative deficit models. The phenotypic model is more commonly used in LTx and considers frailty through five characteristics; shrinking (weight loss), weakness, exhaustion, slowness and low physical

For peer review only - http://bmjopen.bmj.com/site/about/guidelines.xhtml

activity [9]. The cumulative deficit model conceptualises frailty as an accumulation of symptoms, comorbidities, diseases and health deficiencies. The greater the number of "deficits", the higher the frailty of the individual [10]. Although previously linked to decreased survival after LTx [11], the validity of the latter model in predicting other LTx outcomes is unclear [5].

Although psychosocial, nutritional and physical impairments impact post-LTx outcomes (1), the clinical operationalisation of frailty measurement in LTx relates overwhelmingly to the physical domain [5,12,13]. This may be due to the feasibility of data collection, the younger age of LTx candidates or the complexity associated with cumulative deficit models [12,14]. The most commonly reported phenotypic frailty measures collected in clinical practice prior to LTx include the Short Physical Performance Battery (SPPB), and the Fried Frailty Phenotype (FFP) [13,15].

Research studies in LTx reflect this phenotypic approach. Although conceptualisation and investigation of biomarkers and their links to frailty [16] (including in patients with lung disease) has recently been undertaken, the field has lacked established markers or cut-offs, preventing their routine use in the evaluation of frailty for LTx [5,12,13]. The recent development of a LTx specific frailty scale, the Lung Transplant Frailty Scale (LT-FS) and its validation in 342 LTx candidates may help to resolve this [13]. This uses clinically feasible measures of body composition (including appendicular skeletal muscle index (ASMI) – and percent body fat by bioelectrical impedance analysis), research-grade serum biomarkers and clinical laboratory blood markers in addition to measurement of physical function (SPPB and FFP). All models of the scale displayed superior predictive validity for short term mortality and

For peer review only

removal from the LTx waiting list than the FFP or SPPB alone, but long-term outcomes have not yet been evaluated.

Previous reviews have identified physical frailty as being detrimental to morbidity and mortality both before and after LTx [17]. Physical frailty is also associated with an increased risk of readmission after LTx [18]. Transplant teams experience the challenges of identifying patients with the physical and psychological reserve necessary to survive such a demanding peri and postoperative period and to thrive long-term [1,12]. This challenge has been exacerbated by the lack of consensus on the optimal assessment tools for frailty in this field [1], which the LT-FS may help to resolve [13]. Despite the current lack of consensus on tool utility, it is widely agreed that further work is required to establish the effectiveness of interventions to modify physical frailty, improve candidate selection and LTx outcomes [1,13,19].

Exercise and Rehabilitation Prior to Lung Transplantation

Rehabilitation for individuals with lung conditions usually takes the form of pulmonary rehabilitation; an evidence-based programme of exercise interventions and education. It aims to reduce dyspnoea, optimise functional capacity, increase participation, and reduce healthcare costs through exacerbations and hospital admissions [20]. Rehabilitation whilst on the waiting list (sometimes known as prehabilitation) is recommended for all LTx candidates [1]. Rehabilitation after LTx is also a vital component of the recovery programme after surgery and ICU/hospital stay [21]. The course of the SARS COVID-19 pandemic has seen many pulmonary rehabilitation schemes develop virtual or telephone offers alongside a face-to-face programme. Technological advances have also led to the development of digital alternatives [22]. A recent European Society for Organ Transplantation consensus statement

For peer review only - http://bmjopen.bmj.com/site/about/guidelines.xhtml

on prehabilitation for solid organ transplant candidates concluded that “it is feasible, acceptable and safe for adults to participate in exercise, nutritional and pscychosocial interventions during the waiting list period” [23, p.19]. The importance of multimodal programmes was highlighted but the lack of a core outcome set and specific guidance on programme content highlights the need for ongoing, high quality research studies in this field.

Potential Impact of the Intervention

Advancing lung disease impacts on exercise capacity and accelerates muscle atrophy. Reduced muscle mass and quadriceps strength are observed in the pre and post-transplant period, with the impact of post-operative immunosuppressive therapy an important factor in the development of obesity, sarcopenia and osteoporosis [24,25]. A recent systematic review concluded that exercise programmes containing aerobic and resistance training prior to LTx have the capacity to improve exercise capacity and quality of life with some evidence of increases in muscle strength [21]. Multi-component exercise in older adults, has been shown to contribute to an improvement in muscle strength, balance, lung function, physiological processes (such as reduced oxidative damage, inflammation and improved mitochondrial function). and physical activity which in turn impacts on muscle composition and function, strength, function in daily life and therefore physical frailty [26]. Pulmonary rehabilitation has been shown to improve FFP scores towards a more robust state in the short term in individuals with COPD [27].

Why it is Important to do this Review

Professionals in the transplant community are increasingly measuring frailty in attempts to evaluate surgical suitability and weigh the risks and potential benefits of LTx for older individuals. There is emerging evidence linking frailty to reduced quality of life and mortality

For peer review only

For peer review only - http://bmjopen.bmj.com/site/about/guidelines.xhtml

on the lung transplant waiting list and postoperative adverse outcomes including increased length of hospital stay, readmission, disability and worse health related quality of life [5]. Frailty is therefore a significant concern for transplant programmes.

The aim of LTx is to improve survival, function and quality of life of the recipient, whilst considering the ethical elements of who will benefit from such a limited pool of donor organs [12]. Understanding and targeting reversible components of frailty has the potential to improve physical condition ahead of major surgery, reduce waiting list mortality and potentially impact post-operative outcomes and maximise benefit from LTx [4,12,28]. Identifying the best strategies to improve frailty prior to transplantation has been highlighted as an area that requires investigation [4,5,13].

Previous systematic reviews have documented the beneficial effect of rehabilitation on exercise capacity and quality of life prior to a lung transplant [21,29]. However, there are no prior reviews specifically addressing interventions in modifying physical frailty in this population despite the significance of the problem and link to poor outcomes [17].

Aims and Objectives

This systematic review aims to evaluate the impact of exercise interventions in modifying physical frailty for adults awaiting LTx. We also aim to identify any harms that occur as a result of an exercise intervention.

METHODS AND ANALYSIS

Study Design

For peer review only - http://bmjopen.bmj.com/site/about/guidelines.xhtml

This study will be a systematic review with methodology following guidance from the Cochrane Handbook of Systematic Reviews [30]. It will be reported in accordance with the Preferred Reporting Items for Systematic Reviews and Meta-Analyses checklist (PRISMA) [31]. Any important amendments will be documented on Prospero.

Inclusion and Exclusion Criteria

Types of Studies:

Preliminary searches indicated a very few randomised controlled trials (RCT) and non-randomised controlled studies, which we initially planned to include. We therefore widened the inclusion criteria to original primary research studies with any design, including those without controls, with more than 10 participants; this amendment was registered on Prospero. We therefore anticipate including randomised and non-randomised trials, cohort studies and case series. Cross-sectional studies may be eligible if they assess frailty following an intervention. We will not include evidence syntheses, commentaries, case studies, and non-systematic narrative reviews. Where possible, we will extract and report any definitive statements regarding ethical procurement of donor organs and exclude reports from institutions with unethical organ harvesting practices.

For peer review only

Types of Participants:

We will include studies of adults over the age of 18, on a waiting list (candidates) for a single or double LTx. Individuals with any underlying lung disease who have met the criteria to be accepted onto the waiting list at a LTx centre, where frailty has been assessed will be included. There are well established and accepted internationally agreed criteria for LTx [1]. We will include studies involving candidates for primary or subsequent repeat LTx procedures. LTx

For peer review only - http://bmjopen.bmj.com/site/about/guidelines.xhtml

performed by any surgical incision site will be included. Studies of candidates listed for a multi-organ transplant in the same surgical procedure (including but not limited to, concurrent heart and lung or lung and liver) will be excluded unless data is provided for lung recipients only or multi-organ candidates comprise under 25% of study. We will exclude studies of people completing interventions whilst receiving extra corporeal membrane oxygenation (ECMO) as a bridge to transplant; they are restricted to interventions within the intensive care unit which is not the focus of this review.

Types of Intervention

We will include any formal physical exercise or physical activity prescribed under professional guidance which includes formal evaluation of outcomes. The intervention may be supervised or unsupervised, face to face or virtual and performed in any setting (community or hospital). There is no minimum length or intensity of intervention. The intervention may be single (one form of exercise), multimodal (for example a combination of strength and endurance training) or multi-component (e.g. exercise and a nutritional intervention). The intervention must contain an exercise or physical activity component. Physical activity is defined as any bodily movement produced by skeletal muscles that results in energy expenditure. Exercise is defined as a subset of physical activity that is planned, structured and repetitive and has an objective of the improvement or maintenance of physical fitness [32]. Multimodal and multi-component interventions in this population are clinically important due the need to address the complex interaction between the physical and psychological health of the patient prior to transplantation [23].

Types of Comparators

For peer review only - http://bmjopen.bmj.com/site/about/guidelines.xhtml

For peer review only

We will accept any comparator or no comparator. Where studies include comparison groups we anticipate identifying comparisons of rehabilitation with one or more of the following: no intervention, "usual care", advice only, or an alternative intervention which may or may not meet our intervention criteria. The specific rehabilitation intervention should be the only systematic difference between the groups.

Outcomes:

We will consider any validated physical frailty measures as primary outcomes: measures of Phenotypic frailty (FFP) or cumulative deficit frailty models (e.g. Clinical Frailty Scale, Electronic Frailty Index,).

We will also include surrogate markers of physical frailty e.g. SPPB, sarcopenia via hand grip dynamometry, quadriceps force, CT scan (muscle cross sectional area), muscle strength testing of upper of lower limb: manual or non-manual (e.g. 1 rep max), sit to stand testing and objective assessments of balance.

Studies must include at least one direct or indirect measure of frailty, measured before (except in the case of cross-sectional studies) and at any time point after the intervention in order to be eligible for inclusion. We will specify whether outcomes have been assessed pre or postoperatively and discuss any post LTx rehabilitation (as a potential confounder) where relevant. Where no relevant outcome is reported, we will attempt to contact authors of all otherwise relevant studies to establish if any further outcomes were assessed but not reported and, where possible, obtain relevant data. We will distinguish between studies excluded after confirmation that no relevant outcomes were assessed and those where this confirmation was not possible.

For peer review only - http://bmjopen.bmj.com/site/about/guidelines.xhtml

Where studies report a relevant primary outcome, we will consider the following as secondary outcomes: Mortality (on waiting list or post-operatively), hospital/ICU length of stay, health-related quality of life measures. Any adverse events reported during interventions will be recorded.

Search Strategies

Literature search strategies have been developed using medical subject headings (MeSH) related to rehabilitation, exercise and lung transplant candidates with support from an experienced medical librarian. Trials will be identified from searches of the following databases: MEDLINE (Ovid) 1980 to date, EMBASE (Ovid) 1980 to date, CINHAL Plus (EBSCO) 1980 to date, Cochrane Central Register of Controlled Trials (CENTRAL), the Cochrane Library, trials registries (ClinicalTrials.gov and the WHO trials portal). All databases will be searched from 1980 to the present. The success of LTx was established only after the discovery and introduction of the immunosuppressive agent cyclosporine which became accepted practice in the early 1980s [33]. A draft search strategy for MEDLINE is given in Table 1 with this strategy being adopted and modified for other databases (see supplementary material). We will check if abstracts meeting the inclusion criteria have since been published in full text. We will exclude theses or unpublished studies. In an attempt to reduce our susceptibility to reporting bias, we will provisionally include all studies meeting our inclusion criteria for population, intervention, comparator and study design. If frailty outcomes are not reported, we will contact authors to check if any were measured but excluded from the publication. We will not exclude studies based on outcomes in the first instance.

For peer review only - http://bmjopen.bmj.com/site/about/guidelines.xhtml

We will also search the references of all identified included studies and of identified systematic or scoping reviews.

Table 1. Ovid MEDLINE(R) Search strategy

Number of search	Search term
1	lung transplant*.ti,ab.
2	exp Lung Transplantation/
3	1 OR 2
4	(wait* or candidate* or pre op* or pre-op* or await*).ti,ab.
5	Waiting Lists/
6	Preoperative Exercise/ or Preoperative Care/
7	4 or 5 or 6
8	(exercis* or rehab* or prehab*).ti,ab.
9	exp Rehabilitation/
10	8 OR 9
11	3 AND 7 AND 10
12	limit 11 to (yr="1980 -Current" and "all adult (19 plus years)")

For peer review only

Selecting Studies

Following deduplication in Endnote, title and abstract and full text screening will be performed by two independent reviewers (LM and GN) against the pre-defined inclusion criteria using Rayyan software [34]. Any discrepancies between reviewers will be resolved by consensus or consultation with a third reviewer (HH or CT). Any non-English language studies will be retained and listed for reference but not included in the synthesis process. This will

For peer review only - http://bmjopen.bmj.com/site/about/guidelines.xhtml

enhance transparency, mitigate language publication bias and maps the extent of missing evidence in our results.

Data Extraction and Management

Two authors will pilot and agree a data extraction form. Data extraction will be completed by one author (LM) and checked by a second (GN). Any discrepancy will be reconciled by consensus or a third author (HH or CT). The data extraction form will be based on the information collated in Table 2.

Table 2: Data extraction plan.

	**Data to be extracted**
**Study Design**	Design (e.g. RCT)<br>Date of study period and publication<br>Country of origin and setting e.g. single site or multi-centre study<br>Duration of follow-up<br>Sample size calculation<br>Ethical approval and (methods of) informed consent, IRB number.<br>Any statement of ethical procurement of donor organs or reports of donor consent status.
**Participants**	Inclusion/Exclusion Criteria<br>Characteristics for each group including:<br>Number of participants<br>Age (Mean (s.d.))<br>Ratio of male:female<br>Disease/diagnosis<br>Co-morbidities<br>Primary transplant (or repeat transplant after graft failure); number/percentage per group.<br>Characteristics that stratify health opportunities and outcomes (PROGRESS-plus) [35]

For peer review only

For peer review only - http://bmjopen.bmj.com/site/about/guidelines.xhtml

**Interventions**	Based on TIDieR: Template for Intervention Description and Replication Checklist [36] and the Consensus on Exercise Reporting Template (CERT) [37].<br>Type of exercise component or modality /other intervention in each group.<br>Specific exercises and target muscle groups trained.<br>Equipment required or provided.<br>Frequency, intensity and duration of intervention (including how this was prescribed and measured).<br>Location of intervention delivery (e.g. Home, hospital, community, gym)<br>Who delivered the intervention (profession, level of experience, training).<br>Mode of intervention delivery (e.g. Face to face, telephone, video call)<br>Any reported tailoring, modification or personalisation of the intervention.<br>Adherence measurement methods and outcomes (including adherence to attendance and exercise prescription)<br>Details of any motivation or behavioural change strategies used.
**Outcomes and measures of effect**	Outcomes: Any validated measure or surrogate measure of frailty including, but not limited to; FFP, Clinical Frailty Scale, Electronic Frailty Index, SPPB, hand grip strength, measures of muscle strength/size/force, sit to stand testing, measures of balance.<br>Definition for each outcome alongside minimal clinically important difference (MCID) where available, scale limits and direction of benefit.<br>Time points measured.<br>Validation of tool. For each group and each outcome at each time point we will extract the number of participants with data in each group. We will extract post intervention and change from baseline data.<br>Continuous outcomes: mean with s.d. for each group. Medians with IQR will be extracted where reported. Mean differences or standardised mean differences with 95% CI will be extracted where these are the only reported data. P values will be extracted in the absence of other outcome data as will descriptive reporting of results.

For peer review only

	Dichotomous outcomes: number in each group with event. For adverse events types of adverse events will also be reported. Relative risks or odds ratios with 95% CI will be extracted where these are the only reported data.<br>Time to event data: hazard ratios or data to calculate these.
**Other**	Other relevant details of study design or intervention.<br>Funding Source<br>Conflicts of interest<br>Recruitment failure<br>Patient and public involvement or engagement

Where outcomes use continuous scales of measurement (e.g. composite frailty scores, muscle cross-sectional area) we will express the results as the mean difference or, where scales or units are unclear or composites, standardised mean difference (SMD) with 95% confidence intervals (CI). Where outcome measures vary across studies, we will express them as SMD if we consider that they are assessing the same outcome construct. We will express dichotomous outcomes as risk ratios (RR) with 95% CI. Survival outcomes will be reported as hazard ratios with 95% CI. If outcomes are reported at multiple time points, we will extract all the data, but will analyse primary data at the end of the intervention and at the latest time point reported. We anticipate that a mix of final and change from baseline scores will be available and will extract both or either, as mean differences with SD (see data synthesis).

Missing Data:

Any missing data will be requested from the original author by email and included in the review. If we are unable to obtain the missing data, where possible, we will calculate or impute these (e.g. where measures of variance are not available). Where outcome data

For peer review only - http://bmjopen.bmj.com/site/about/guidelines.xhtml

remain missing and cannot be imputed we will use assumptions in analysing dichotomous data; that is we will assume that missing participants did not have the outcome assessed but we will explore the impact of this assumption with a sensitivity analysis. For continuous outcomes we will perform a completed case analysis and will not attempt to impute results for missing participants.

Risk of Bias Assessment

RCTs and quasi-RCTs will be assessed using the Cochrane Risk of Bias tool (RoB2) with an extension for cluster or crossover RCTs as required [30]. ROBINS-I [38] or another appropriate tool will be used for non-randomised controlled studies. Other study designs (e.g. cohort and cross-sectional studies and case-series) will be assessed using an appropriate tool. Risk of bias assessments will be done by two reviewers independently (LM and GN) and any discrepancies reconciled by a 3rd reviewer (HH or CT).

Data analysis - Strategy for Data Synthesis

We will take a pragmatic approach to data management and synthesis based on the data available and the designs of included studies. Clinical and methodological heterogeneity will be considered initially. If sufficient studies are identified with low heterogeneity, statistical heterogeneity will be considered to determine if a random-effects meta-analysis should be performed. We will not combine randomised and non-randomised studies in the same analysis but may display them using a single forest plot. We anticipate that we will identify studies reporting one or both of post-intervention data and change from baseline data. Decisions on which of these we use in analyses will be guided by pragmatic considerations; while we may combine the two types of data if mean differences are calculated we will not do so if SMDs are used. We will fully document these choices. If change from baseline data is

For peer review only - http://bmjopen.bmj.com/site/about/guidelines.xhtml

presented without final score data, we will use an approach suggested in the Cochrane handbook [29] to estimate the SD of the final score using an estimated correlation coefficient.

If the studies identified vary widely in terms of population, study design, intervention and outcomes a meta-analysis may be inappropriate and therefore a narrative synthesis of findings will be produced and reported following the Synthesis without Meta-analysis (SWiM) guidelines for narrative evidence synthesis [39]. If we are unable to perform meta-analysis we will present studies where we can compute a comparable effect size on a forest plot without combination. Where it is not possible to do this, we will report the variety of reported effect sizes in a table with confidence intervals and may also display effect directions.

Assessment of heterogeneity

We will initially assess statistical heterogeneity by visual inspection of the forest plot followed by a quantification using the I^2 statistic. (75% - 100% considerable heterogeneity; 50-90% considered to represent substantial heterogeneity; 30-60% moderate and 0-40% considered unimportant heterogeneity) [30].

Subgroup analysis

Participant heterogeneity may be investigated using subgroup analysis by disease type (COPD, CF, IPF for example). Subgroup analysis by age may be considered (18-69, and ≥70 years) as lung transplant recipients over the age of 70 years have a decreased longer-term survival [40]. Intervention heterogeneity may be investigated by subgroup analysis including the following potential groups: virtual and in person rehabilitation, exercise intensity, exercise type (e.g. aerobic versus resistance) or the degree of supervision (e.g. fully supervised versus non

For peer review only - http://bmjopen.bmj.com/site/about/guidelines.xhtml

supervised). We will exercise caution in implementing these prespecified analyses and are mindful of the risks of performing multiple such analyses.

Sensitivity analysis

If appropriate we will use sensitivity analyses to explore the impact of any imputation of or assumptions about missing data. If we perform a meta-analysis with very few studies we will conduct a fixed effects analysis to assess the impact of the random effects model in this context [41].

Summary of Findings tables.

We will present a summary of the main findings of the review in a table format including key information regarding the magnitude of effects for each outcome alongside a rating of the certainty of evidence. We will use the Grading of Recommendations Assessment, Development and Evaluation working group methodology considerations (risk of bias, consistency of effect, imprecision, indirectness and publication bias) to assess the quality of the body of evidence for each prespecified outcome [42,43]. We will justify all decisions to down- or up-grade the certainty of the evidence. If appropriate we will present the evidence in summary of findings tables generated using GRADEpro software [44].

Patient and Public Involvement

Local lung transplant support group attendees have been involved in some preliminary patient and public engagement work prior to the data collection for this study. Further PPI work will be completed to contribute to the production of a plain language summary and insight into dissemination strategies.

For peer review only - http://bmjopen.bmj.com/site/about/guidelines.xhtml

ETHICS AND DISSEMINATION

Ethical approval is not required because this is a systematic review using previously published data. We are mindful of the ethical considerations relating to the generation of this data and will record relevant information relating to this.

This systematic review has been registered prospectively (registration number CRD42022363730). Its findings will be submitted to international transplantation conferences and peer reviewed journals for dissemination of the results and conclusions. Results, and their implications will be shared at local and national meetings of LTx clinicians. We will also share findings with people living with chronic lung disease on the transplant waiting list and their carers via local patient support groups.

STATEMENTS OF FINANCIAL SUPPORT

LM holds a part-time predoctoral fellowship funded by the National Institute for Health and Care Research Applied Research Collaboration Greater Manchester (NIHR ARC-GM) (Grant award number NIHR200174). CT is a CI and partially funded and GN is wholly funded by NIHR ARC-GM. The views expressed in this publication are those of the authors and not necessarily those of the National Institute for Health and Care Research or the Department of Health and Social Care or its partner organisations.

Contributions: LM had the idea for the review, wrote the first draft of the protocol and designed the search strategy; GN, HH and CT advised on the protocol, edited and commented substantively on the protocol; all authors approved the manuscript prior to submission.

Competing Interests Declaration: The authors have no competing interests to declare.

ACKNOWLEDGEMENTS:

For peer review only - http://bmjopen.bmj.com/site/about/guidelines.xhtml

The authors thank Bethan Morgan (librarian at Manchester University NHS Foundation Trust) for her support with electronic search strategies for this review and Sarah Rhodes (medical statistician) for her support with the meta-analysis approach.

REFERENCE LIST

1. Leard LE, Holm AM, Valapour M, et al. Consensus document for the selection of lung transplant candidates: An update from the International Society for Heart and Lung Transplantation. J Heart Lung Transplant. 2021;40(11):1349-79.
2. NHSBT Annual report of cardiothoracic organ transplantation [website] London (UK) 2020 [Updated 2020, cited March 2023]. Available from https://nhsbtdbe.blob.core.windows.net/umbraco-assets-corp/19874/nhsbt-annual-report-on-cardiothoracic-organ-transplantation-201920.pdf
3. Venado A, McCulloch C, Greenland JR, et al. Frailty trajectories in adult lung transplantation: A cohort study. The Journal of Heart and Lung Transplantation. 2019;38(7):699-707.
4. Kobashigawa J, Dadhania D, Bhorade S, et al. Report from the American Society of Transplantation on frailty in solid organ transplantation. American Journal of Transplantation. 2019;19(4):984-94.
5. Varughese R, Rozenberg D, Singer LG. An update on frailty in lung transplantation. Current Opinion in Organ Transplantation. 2020;25(3):274-9.
6. Valapour M, Lehr CJ, Skeans MA, et al. OPTN/SRTR 2018 Annual Data Report: Lung. American Journal of Transplantation. 2020;20(s1):427-508.

For peer review only - http://bmjopen.bmj.com/site/about/guidelines.xhtml

7. Courtwright A, Cantu E. Lung transplantation in elderly patients. J Thorac Dis. 2017;9(9):3346-51

8. Clegg A, Young J, Iliffe S, et al. Frailty in elderly people. Lancet. 2013;381(9868):752-62.

9. Fried LP, Tangen CM, Walston J, et al. Frailty in older adults: evidence for a phenotype. J Gerontol A Biol Sci Med Sci. 2001;56(3):M146-56.

10. Rockwood K, Mitnitski A. Frailty in Relation to the Accumulation of Deficits. The Journals of Gerontology: Series A. 2007;62(7):722-7.

11. Wilson ME, Vakil AP, Kandel P, et al. Pretransplant frailty is associated with decreased survival after lung transplantation. J Heart Lung Transplant. 2016;35(2):173-8.

12. Agarwal A, Neujahr DC. Frailty in lung transplantation: Candidate assessment and optimization. Transplantation. 2021;105(10):2201-12.

13. Singer JP, Christie JD, Diamond JM, et al. Development of the Lung Transplant Frailty Scale (LT-FS). J Heart Lung Transplant. 2023;42(7):892-904.

14. Spiers GF, Kunonga TP, Hall A, et al. Measuring frailty in younger populations: a rapid review of evidence. BMJ Open. 2021;11(3):e047051.

15. Snyder LD, Singer LG. Beyond the eyeball test: Measures of frailty in lung transplantation. The Journal of Heart and Lung Transplantation. 2019;38(7):708-9.

16. Hakeem FF, Maharani A, Todd C, et al. Development, validation and performance of laboratory frailty indices: A scoping review. Archives of Gerontology and Geriatrics. 2023;111:104995.

17. Montgomery E, Macdonald PS, Newton PJ, et al. Frailty in lung transplantation: a systematic review. Expert Review of Respiratory Medicine. 2020;14(2):219-27.

For peer review only - http://bmjopen.bmj.com/site/about/guidelines.xhtml

18. Courtwright AM, Zaleski D, Gardo L, et al. Causes, Preventability, and Cost of Unplanned Rehospitalizations Within 30 Days of Discharge After Lung Transplantation. Transplantation. 2018;102(5):838-44

19. Wilson ME, Vakil AP, Kandel P, et al. Pretransplant frailty is associated with decreased survival after lung transplantation. J Heart Lung Transplant. 2016;35(2):173-8.

20. Holland AE, Cox NS, Houchen-Wolloff L, et al. Defining Modern Pulmonary Rehabilitation. An Official American Thoracic Society Workshop Report. Ann Am Thorac Soc. 2021;18(5):e12-e29.

21. Hume E, Ward L, Wilkinson M, et al. Exercise training for lung transplant candidates and recipients: A systematic review. European Respiratory Review. 2020;29(158):1-19.

22. Singer JP, Soong A, Bruun A, et al. A mobile health technology enabled home-based intervention to treat frailty in adult lung transplant candidates: A pilot study. Clin Transplant. 2018;32(6):e13274.

23. Annema C, De Smet S, Castle EM, Overloop Y, Klaase JM, Janaudis-Ferreira T, et al. European Society of Organ Transplantation (ESOT) Consensus Statement on Prehabilitation for Solid Organ Transplantation Candidates. Transplant International. 2023;36

24. Rozenberg D, Wickerson L, Singer LG, Mathur S. Sarcopenia in lung transplantation: a systematic review. J Heart Lung Transplant. 2014;33(12):1203-12.

25. Reinsma GD, ten Hacken NH, Grevink RG, van der Bij W, Koëter GH, van Weert E. Limiting factors of exercise performance 1 year after lung transplantation. J Heart Lung Transplant. 2006;25(11):1310-6.

For peer review only

For peer review only - http://bmjopen.bmj.com/site/about/guidelines.xhtml

26. Angulo J, El Assar M, Álvarez-Bustos A, et al. Physical activity and exercise: Strategies to manage frailty. Redox Biol. 2020;35:101513.

27. Maddocks M, Kon SSC, Canavan JL, et al. Physical frailty and pulmonary rehabilitation in COPD: a prospective cohort study. Thorax. 2016;71(11):988-95.

28. Rozenberg D, Orsso CE, Chohan K, et al. Clinical outcomes associated with computed tomography-based body composition measures in lung transplantation: a systematic review. Transpl Int. 2020;33(12):1610-25.

29. Hoffman M, Chaves G, Ribeiro-Samora GA, et al. Effects of pulmonary rehabilitation in lung transplant candidates: a systematic review. BMJ open. 2017 Feb 1;7(2):e013445.

30. Higgins JPT, Thomas J, Chandler J, et al. (editors)[website]. Cochrane Handbook for Systematic Reviews of Interventions version 6.3 (updated February 2022). Cochrane, 2022. Available from www.training.cochrane.org/handbook

31. Page MJ, McKenzie JE, Bossuyt PM, et al. The PRISMA 2020 statement: an updated guideline for reporting systematic reviews. BMJ. 2021;372:n71.

32. Casperson CJ, Powell KE, Christenson GM. Physical activity, exercise and physical fitness: Definitions and distinctions for health-related research. Public Health Reports. 1985;100(2):126-31.

33. Venuta F, Van Raemdonck D. History of lung transplantation. J Thorac Dis. 2017;9(12):5458-71.

34. Ouzzani M, Hammady H, Fedorowicz Z, et al. Rayyan—a web and mobile app for systematic reviews. Systematic Reviews. 2016;5(1):210.

35. O'Neill J, Tabish H, Welch V, et al. Applying an equity lens to interventions: using PROGRESS ensures consideration of socially stratifying factors to illuminate inequities in health. Journal of Clinical Epidemiology. 2014;67(1):56-64.

For peer review only - http://bmjopen.bmj.com/site/about/guidelines.xhtml

36. Hoffmann TC, Glasziou PP, Boutron I, et al. Better reporting of interventions: template for intervention description and replication (TIDieR) checklist and guide. BMJ : British Medical Journal. 2014;348:g1687.

37. Slade SC, Dionne CE, Underwood M, Buchbinder R, Beck B, Bennell K, et al. Consensus on Exercise Reporting Template (CERT): Modified Delphi Study. Physical Therapy. 2016;96(10):1514-24

38. Sterne JAC, Hernán MA, Reeves BC, et al. ROBINS-I: a tool for assessing risk of bias in non-randomised studies of interventions. BMJ. 2016;355:i4919.

39. Campbell M, McKenzie JE, Sowden A, et al. Synthesis without meta-analysis (SWiM) in systematic reviews: reporting guideline. BMJ. 2020;368:l6890.

40. Hayanga AJ, Aboagye JK, Hayanga HE, et al. Contemporary analysis of early outcomes after lung transplantation in the elderly using a national registry. J Heart Lung Transplant. 2015;34(2):182-8.

41. Bender R, Friede T, Koch A, et al. Methods for evidence synthesis in the case of very few studies. Research Synthesis Methods. 2018;9(3):382-92.

42. Guyatt G, Oxman AD, Akl EA, et al. GRADE guidelines: 1. Introduction—GRADE evidence profiles and summary of findings tables. Journal of Clinical Epidemiology. 2011;64(4):383-94.

43. Schünemann H, Brożek J, Guyatt G, Oxman A. GRADE handbook for grading quality of evidence and strength of recommendations. guidelinedevelopment.org/handbook2013

44. GRADEpro GDT: GRADEpro Guideline Development Tool [Software]. McMaster University and Evidence Prime, 2022. Available from gradepro.org

For peer review only - http://bmjopen.bmj.com/site/about/guidelines.xhtml

For peer review only

For peer review only - http://bmjopen.bmj.com/site/about/guidelines.xhtml

For peer review only

For peer review only - http://bmjopen.bmj.com/site/about/guidelines.xhtml

Supplementary Materials – Database search strategies

Search strategy for World Health Organisation—International clinical trials registration platform	
**Number of search**	**Search term**
1	Frail*
2	Lung transplant* AND (candidate OR wait* OR list)
3	Exercise OR prehab* OR rehab*
4	1 AND 2 AND 3

Search strategy for ClinicalTrials.gov	
**Number of search**	**Search term**
Condition or disease	Frail OR frailty OR frailness
	Lung transplant OR lung transplantation OR transplantation of lung OR transplant OR grafting procedure OR transplantation OR transplanted OR transplanting
	Lung OR pulmo*
	Rehabilitation OR rehab OR rehabilitative
Study Type	All studies
Study Results	All studies
Eligibility Criteria	Adult (18-64)<br>Sex (all)

Search strategy for CINAHL	
**Number of search**	**Search term**
1	TI lung transplant* OR AB lung transplant*
2	MH (“lung transplantation+”)
3	1 OR 2
4	TI (wait* or candidate* or pre op* or await*) OR AB (wait* or candidate* or pre op* or await*)
5	(MH “waiting lists”) OR (MH “pretransplantation period”)
6	(MH “Preoperative care+”)
7	4 OR 5 OR 6
8	TI (exercise* or rehab* or prehab*) OR AB (exercise* or rehab* or prehab*)
9	(MH “rehabilitation+”)
10	8 OR 9
11	3 AND 7 AND 10

For peer review only

Search strategy for Cochrane

Number of search	Search term
1	(lung transplant*):ti,ab,kw (Word variations have been searched)
2	MeSH descriptor: [Lung Transplantation] explode all trees
3	1 OR 2
4	((wait* or candidate* or pre op* or pre-op* or await*)):ti,ab,kw (Word variations have been searched)
5	MeSH descriptor: [Waiting Lists] explode all tree
6	MeSH descriptor: [Preoperative Exercise] explode all trees
7	MeSH descriptor: [Preoperative Care] this term onl
8	4 OR 5 OR 6 OR 7
9	((exercis* or rehab* or prehab*)):ti,ab,kw (Word variations have been searched)
10	MeSH descriptor: [Rehabilitation] explode all trees
11	9 OR 10
12	3 AND 8 AND 11

For peer review only

Search strategy for EMBASE (OVID)

Number of search	Search term
1	lung transplant*.ti,ab
2	exp Lung Transplantation/
3	1 or 2
4	(wait* or candidate* or pre op* or pre-op* or await*).ti,ab.
5	Waiting Lists/
6	Preoperative Exercise/ or Preoperative Care/
7	4 or 5 or 6
8	(exercis* or rehab* or prehab*).ti,ab.
9	exp Rehabilitation/
10	8 or 9
11	3 AND 7 AND 10
12	limit 11 to (yr="1980 -Current" and (adult <18 to 64 years> or aged <65+ years>))

PRISMA-P (Preferred Reporting Items for Systematic review and Meta-Analysis Protocols) 2015 checklist: recommended items to address in a systematic review protocol*

Section and topic	Item No	Checklist item	Information reported?	Page number
**ADMINISTRATIVE INFORMATION**				
Title:		Rehabilitation interventions to modify physical frailty in adults before lung transplantation: A systematic review protocol		
Identification	1a	Identify the report as a protocol of a systematic review	Yes	1
Update	1b	If the protocol is for an update of a previous systematic review, identify as such	n/a	n/a
Registration	2	If registered, provide the name of the registry (such as PROSPERO) and registration number	Yes	2
Authors:				
Contact	3a	Provide name, institutional affiliation, e-mail address of all protocol authors; provide physical mailing address of corresponding author	Yes	Submitted to journal
Contributions	3b	Describe contributions of protocol authors and identify the guarantor of the review	Yes	22
Amendments	4	If the protocol represents an amendment of a previously completed or published protocol, identify as such and list changes; otherwise, state plan for documenting important protocol amendments	Yes (plan for reporting amendments)	8
Support:				
Sources	5a	Indicate sources of financial or other support for the review	Yes	22
Sponsor	5b	Provide name for the review funder and/or sponsor	Yes	22
Role of sponsor or funder	5c	Describe roles of funder(s), sponsor(s), and/or institution(s), if any, in developing the protocol	Yes	22
**INTRODUCTION**				
Rationale	6	Describe the rationale for the review in the context of what is already known	Yes	6-7
Objectives	7	Provide an explicit statement of the question(s) the review will address with reference to participants, interventions, comparators, and outcomes (PICO)	Yes	7
**METHODS**				
Eligibility criteria	8	Specify the study characteristics (such as PICO, study design, setting, time frame) and report characteristics (such as years considered, language, publication status) to be used as criteria for eligibility for the review	Yes	8-11
Information sources	9	Describe all intended information sources (such as electronic databases, contact with study authors, trial registers or other grey literature sources) with planned dates of coverage	Yes	11,14.

For peer review only

Search strategy	10	Present draft of search strategy to be used for at least one electronic database, including planned limits, such that it could be repeated	Yes	11 (and supplementary info)
Study records:				
Data management	11a	Describe the mechanism(s) that will be used to manage records and data throughout the review	Yes	12
Selection process	11b	State the process that will be used for selecting studies (such as two independent reviewers) through each phase of the review (that is, screening, eligibility and inclusion in meta-analysis)	Yes	12
Data collection process	11c	Describe planned method of extracting data from reports (such as piloting forms, done independently, in duplicate), any processes for obtaining and confirming data from investigators	Yes	12,14
Data items	12	List and define all variables for which data will be sought (such as PICO items, funding sources), any pre-planned data assumptions and simplifications	Yes	12-16 Table 2
Outcomes and prioritization	13	List and define all outcomes for which data will be sought, including prioritization of main and additional outcomes, with rationale	Yes	10 Table 2 – page 13
Risk of bias in individual studies	14	Describe anticipated methods for assessing risk of bias of individual studies, including whether this will be done at the outcome or study level, or both; state how this information will be used in data synthesis	Yes	15-17
Data synthesis	15a	Describe criteria under which study data will be quantitatively synthesised	Yes	15-16
	15b	If data are appropriate for quantitative synthesis, describe planned summary measures, methods of handling data and methods of combining data from studies, including any planned exploration of consistency (such as I^2, Kendall's τ)	Yes	15-16
	15c	Describe any proposed additional analyses (such as sensitivity or subgroup analyses, meta-regression)	Yes	16
	15d	If quantitative synthesis is not appropriate, describe the type of summary planned	Yes	
Meta-bias(es)	16	Specify any planned assessment of meta-bias(es) (such as publication bias across studies, selective reporting within studies)	n/a	Selective reporting: page 10
Confidence in cumulative evidence	17	Describe how the strength of the body of evidence will be assessed (such as GRADE)	Yes	16-17

*** It is strongly recommended that this checklist be read in conjunction with the PRISMA-P Explanation and Elaboration (cite when available) for important clarification on the items. Amendments to a review protocol should be tracked and dated. The copyright for PRISMA-P (including checklist) is held by the PRISMA-P Group and is distributed under a Creative Commons Attribution Licence 4.0.**

For peer review only

From: Shamseer L, Moher D, Clarke M, Ghersi D, Liberati A, Petticrew M, Shekelle P, Stewart L, PRISMA-P Group. Preferred reporting items for systematic review and meta-analysis protocols (PRISMA-P) 2015: elaboration and explanation. BMJ. 2015 Jan 2;349(jan02 1):g7647.

For peer review only
